# A COVID-19 field hospital in a conference centre – The Cape Town, South Africa experience

**DOI:** 10.4102/phcfm.v13i1.3140

**Published:** 2021-12-09

**Authors:** Bojana Bulajic, Kamlin Ekambaram, Colleen Saunders, Vanessa Naidoo, Lee Wallis, Nabeela Amien, Tasleem Ras, Klaus von Pressentin, Gamuchirai Tadzimirwa, Nadia Hussey, Steve Reid, Peter Hodkinson

**Affiliations:** 1 Division of Emergency Medicine, Faculty of Health Sciences, University of Cape Town, Cape Town, South Africa; 2 Division of Family Medicine, Faculty of Health Sciences, University of Cape Town, Cape Town, South Africa; 3 Division of Clinical Haematology, Department of Medicine, Faculty of Health Sciences, University of Cape Town, Cape Town, South Africa; 4 Department of Internal Medicine, Faculty of Health Sciences, University of Cape Town, Cape Town, South Africa; 5 Primary Health Care Directorate, Faculty of Health Sciences, University of Cape Town, Cape Town, South Africa

**Keywords:** COVID, field hospital, multidisciplinary, palliative care, South Africa, health services, pandemic, infectious diseases

## Abstract

**Background:**

The coronavirus pandemic has put extreme pressure on health care services in South Africa.

**Aim:**

To describe the design, patients and outcomes of a field hospital during the first wave of the coronavirus disease 2019 (COVID-19) pandemic.

**Setting:**

The Cape Town International Convention Centre was the first location in Cape Town to be commissioned as a field hospital that would serve as an intermediate care bed facility.

**Methods:**

This was a retrospective descriptive study of patients admitted to this facility between 8th June 2020 and 14th August 2020 using deidentified data extracted from patient records.

**Results:**

There were 1502 patients admitted, 56.4% female, with a mean age of 58.6 years (standard deviation [s.d.]: 14.2). The majority of patients (82.9%) had at least one comorbidity, whilst 15.4% had three or more. Nearly 80.0% (79.8%) of patients required oxygen and 63.5% received steroids, and only 5.7% of patients were required to be transferred for escalation of care. The mean length of stay was 6 days (s.d.: 4.8) with an overall mortality of 5.7%.

**Conclusion:**

This study highlights the role of a field hospital in providing surge capacity. Its use halved the predicted duration of stay at acute care hospitals, allowing them the capacity to manage more unstable and critical patients. Adaptability and responsivity as well as adequate referral platforms proved to be crucial. Daily communication with the whole health care service platform was a critical success factor. This study provides information to assist future health planning and strategy development in the current pandemic and future disease outbreaks.

## Introduction

As the first wave of coronavirus disease 2019 (COVID-19), caused by infection with the severe acute respiratory syndrome coronavirus 2 (SARS-CoV-2), spread rapidly worldwide, the need for additional hospital bed capacity had become a national emergency.^1^ The first case of COVID-19 in South Africa was confirmed on 5th March 2020, followed swiftly by the declaration of a national state of disaster.^2^ Modelling of the epidemic based on European, World Health Organisation (WHO) and Chinese Centre for Disease Control and Prevention data suggested that the Western Cape province could experience a surge of approximately 32 000 cases of moderate to severe COVID-19 requiring hospitalisation at the peak of the first wave,^3^ and subsequently a significant deficit of inpatient beds within the Cape metropole.^4^ Globally, the shortage of hospital beds led to the construction of ‘field hospitals’, serving a variety of purposes ranging from shelters for isolation of mild COVID-19 patients to more specialised COVID-19 hospitals including intensive care beds, and post-acute care centres.^5,6^ Considering the disease profile, bed demand, available infrastructure and time constraints, the most urgent health care need in the Western Cape was identified as intermediate level in-patient care for confirmed COVID-19 positive patients who would require oxygen supplementation, but not ventilation, with an emphasis on family and emergency medicine principles, in line with intermediate care elsewhere.^4^ The Western Cape Government Metro Health Services response strategy, therefore, included the expansion of COVID-19 hospital bed capacity in the form of intermediate care bed facilities (ICBF), the first and largest of which was the Cape Town International Convention Centre (CTICC) ICBF. Patients were only accepted from existing acute care hospitals which provided initial management and stabilisation of acutely ill COVID-19 patients and critical care services where applicable.^7,8^

**FIGURE 1:**
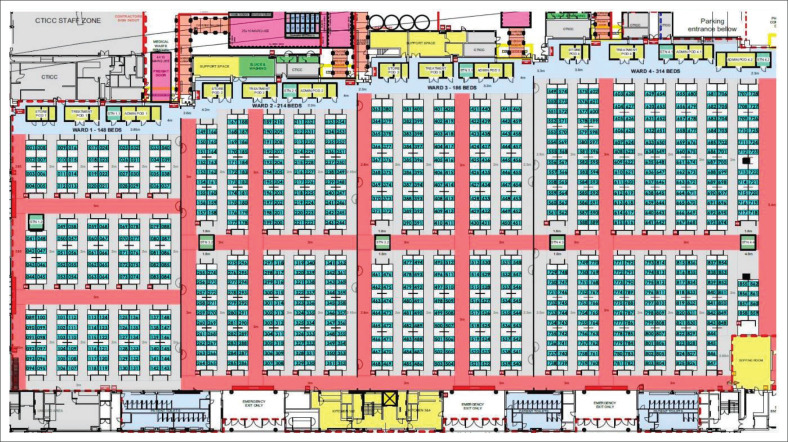
Cape Town International Convention Centre intermediate care bed facility layout.

The CTICC ICBF (Figure 1) served to provide treatment, rehabilitation, disease monitoring, referral and palliative care for patients with COVID-19. This study aims to describe the clinical characteristics, management and outcomes of patients admitted to the CTICC ICBF during the first wave of the COVID-19 pandemic in South Africa. Insights from this study may inform future health systems planning with respect to similar intermediate care facilities in response to pandemic situations.

## Research methods and design

### Study setting

Planning began in March 2020, when four exhibition halls of the CTICC (total floor area 12 683 m^2^) were identified for conversion into an 863-bed inpatient facility, with input and involvement from emergency medicine, disaster management and family medicine experts. Essential medical infrastructure included the installation of a 58-ton bulk oxygen supply to provide piped oxygen to each bed, including 12 beds with capacity for high flow nasal oxygen (HFNO) delivery. Construction of additional areas for donning and doffing of personal protective equipment, doctors’ and nurses’ stations, treatment rooms, mobile X-ray capacity, pharmacy and consumable stores, patient and staff ablutions and medical waste management facilities were required. Protection of staff was paramount in the planning and execution phases of the CTICC ICBF. Infection prevention and control and COVID-19 specific precautions training was provided to all staff and purpose-built donning and doffing facilities were used. Personal protective equipment (surgical scrubs, disposable gown, mask, visor, head cap, gloves) were mandatory on the clinical floor.

The CTICC ICBF was staffed by a multidisciplinary team of 71 doctors (predominantly medical officers led by specialists in emergency medicine, family medicine and internal medicine, as well as a delegation of Cuban doctors), 203 nurses and 40 allied health care workers including physiotherapists, dieticians, pharmacists, radiographers and social workers. The management staffing comprised a facility manager, five clinical managers, a nursing manager, nine operational managers, an administrative manager as well as various administrative support staff. The 173 ancillary staff included an infection prevention and control officer, cleaners, admin clerks, porters and security. The facility was designed and staffed to receive up to 100 admissions per day, with a projected four-day length of stay (LOS) for the majority of patients based on initial modelling.

### Study design

A retrospective record review was performed to describe all patients admitted to the CTICC ICBF for the duration of its functioning, from 8th June 2020 to 14th August 2020. Patients were referred to the facility via an existing online electronic platform already integrated into the Western Cape referral pathways,^9^ and screened by senior clinicians against pre-established admission criteria (Table 1). Admission criteria were dynamic in response to changing acute hospital bed pressure. Patients were accepted from district, regional and tertiary hospitals (hereafter referred to as index facilities), as well as through a newly established telemedicine service in the Cape Metropole. The telemedicine service was an initiative by the Western Cape Department of Health to identify high-risk COVID-19 diabetic patients and offer them admission to intermediate care facilities for diabetic control and disease monitoring.^10^

**TABLE 1:** Admission and discharge criteria for patient flow management at the Cape Town International Convention Centre intermediate care bed facility.

Admission criteria Adult patients: 18 years and older COVID-19 PCR positive or presumptive clinical diagnosis Normal level of consciousness Generally mobile with limited assistance (except for patients referred for palliation) Demonstrated clinical stability for > 48 h at index hospital All relevant investigations completed and organ function stable or improving Classified as appropriate to only receive supportive medical care; or to receive palliative care All criteria need to be fulfilled Discharge criteria Resolution of respiratory symptoms and off supplemental oxygen for > 24 h Minimal oxygen desaturation on exertion Fit for isolation at isolation facility or able to self-isolate Comorbidities controlled All criteria need to be fulfilled Criteria for transfer from intermediate care to acute hospitals and or critical care services Patients in need of escalation of care because of increasing oxygen requirements or worsening respiratory failure Patients requiring intubation and mechanical ventilation, and subsequent transfer to critical care Patients in need of investigations not available on site (e.g. CT chest) Patients who develop acute psychiatric/behavioural symptoms Patients who develop complications that require specialist intervention (e.g. acute abdomen) Any single criterion to be fulfilled

*Source:* Adapted from COVID-19 Intermediate Care Bed Facilities Clinical Care Plan. May 2020. Western Cape: Department of Health.

COVID-19, coronavirus disease 2019; CT, computed tomography; PCR, polymerase chain reaction.

### Study population and sampling strategy

**FIGURE 2:**
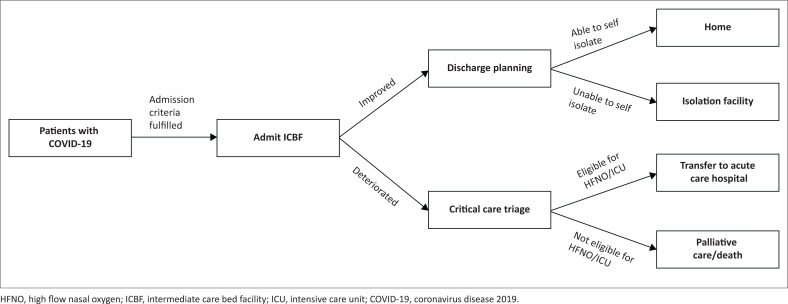
Patient flow through the Cape Town International Convention Centre intermediate care bed facility.

All admissions were included in the study. Patients satisfying the admission criteria arrived at the facility via ambulance. No walk-ins or non-referred patients were permitted. Triage and clerking took place in the admissions area where patients were allocated ward beds based on disease severity, oxygen requirement and acuity of nursing care required. A comprehensive multidisciplinary team, led by specialists in internal medicine, family medicine and emergency medicine, ensured that patients received optimal ward-based care, as well as care from allied health professions (physiotherapy, social workers, dieticians) with a team approach to decision-making and discharge planning. Medical officers were divided into teams under the supervision of a specialist emergency, family or internal medicine physician. Each team was allocated specific areas of beds to ensure continuity of care. All doctors were involved in the management of severely ill/deteriorating patients including training and the use of HFNO. Emergency and family medicine doctors experienced a change of role from seeing mostly outpatients to now managing inpatients in a ward setting, as well as more interdisciplinary discussions and learning from one another. There was also more cohesion and appreciation for other disciplines, including nursing, social work, physiotherapy and pharmacy staff. Communication with patients’ families was a key element, with telephonic and video updates facilitated by devices powered by free Wi-Fi available to staff and patients. Patients were discharged home or to isolation facilities once discharge criteria were met (Table 11^11^), whilst those requiring escalation of care were transferred to designated acute care hospitals. The Western Cape Provincial Critical Care Triage and Decision Tool^12^ was used to determine the eligibility and priority for mechanical ventilatory support. Figure 2 illustrates the flow of patients through the CTICC ICBF. As the first COVID-19 wave subsided, a phased closure of the CTICC ICBF was implemented. A smaller capacity facility then assumed the role of the primary ICBF, culminating in the permanent closure and decommissioning of the CTICC ICBF on 14th August 2020.

### Data collection and analysis

All patient records were digitised and captured on a purpose-built database. Deidentified data were extracted from clinical records by facility clinicians and captured onto an Microsoft Excel spreadsheet for analysis. Extracted data included demographic characteristics, baseline vital signs (including saturation with and without supplemental oxygen), comorbidities, Clinical Frailty Scale^13^ and prescription records. Additional data on outcome, LOS, complications and need for transfer (escalation of care) were also collected. Continuous data were described by mean and standard deviation (s.d.), or median and interquartile range (IQR) as appropriate. Categorical variables were summarised by frequency rates and percentages.

### Ethical considerations

This study was approved by the University of Cape Town Human Research Ethics Committee (HREC Ref 502/2020, R024/2020 and R031/2020), as well as the Western Cape Department of Health Research Committee.

## Results

### Patient demographics and clinical characteristics

**FIGURE 3:**
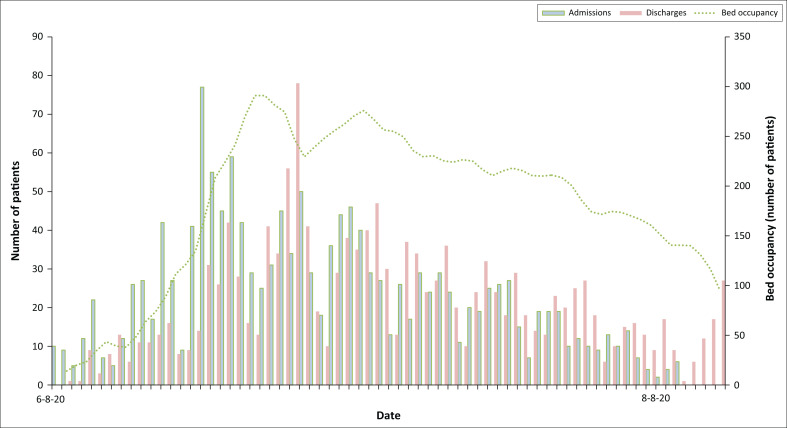
Cape Town International Convention Centre intermediate care bed facility patient turnover.

A total of 1878 electronic referrals were screened, resulting in 1502 patients admitted over the 10 weeks between 8th June and 14th August 2020. A total of 376 patient referrals were declined because they did not meet the ICBF admission criteria, more specifically these patients were better suited for an isolation facility or had deteriorated before transfer. Of the 1502 admitted patients, 56 patient records were excluded from the current analysis because of missing or incomplete medical data. The final sample, therefore, consisted of 1446 patients. The median number of daily admissions was 19 (IQR: 10–29) patients. Admissions peaked in the last week of June with an average of 50 admissions per day between the 22nd June 2020 and 29th June 2020 (Figure 3). This peak coincided with the first wave peak of cases in the Western Cape province. The median daily bed occupancy was 138 (IQR: 71–202) patients, with a maximum occupancy of 277 patients on 28th June 2020 equating to 32% of the total bed capacity.

The mean patient age was 58.6 (s.d. 14.2) years and 56.4% were female (Table 2). The majority (*n* = 1199, 82.9%) had at least one comorbidity, whilst 15.4% (*n* = 223) had three or more. The most common comorbidities were hypertension (59.2%) and diabetes (46.7%). Of note, 84.9% of admitted diabetics had a glycated haemoglobin (HBA1c) HbA1c greater than 7%.

**TABLE 2:** Patient demographics and clinical characteristics on admission (*n* = 1446).

Variables	Mean	s.d.	Median	IQR	*n*	%
**Age (years)**	58.6	14.2	-	-	-	-
≤ 44	-	-	-	-	237	16.4
45–59	-	-	-	-	500	34.4
60–74	-	-	-	-	538	37.2
≥ 75	-	-	-	-	171	11.8
**Sex**						
Female	-	-	-	-	816	56.4
Male	-	-	-	-	630	43.6
**Index (referring) facility**						
District hospital	-	-	-	-	726	50.2
Regional hospital	-	-	-	-	133	9.2
Tertiary hospital	-	-	-	-	473	32.7
Telemedicine/Community diabetics	-	-	-	-	62	4.4
Retirement/frail care facilities	-	-	-	-	47	3.3
Other	-	-	-	-	5	0.4
**Patient descriptors**						
Duration of symptoms (days)	7.0	4.9	-	-	-	-
Clinical frailty scale, median (IQR)	-	-	3	2–4	-	-
Oxygen requirement on referral	-	-	-	-	-	-
Room air	-	-	-	-	216	14.9
Nasal cannula	-	-	-	-	783	54.2
Face mask	-	-	-	-	272	18.8
Non-rebreather mask	-	-	-	-	146	10.1
Non-rebreather/face mask + nasal cannula	-	-	-	-	29	2.0
Saturation on room air (%)	88.3	8.0	-	-	-	-
Saturation on oxygen (%)	95.2	4.7	-	-	-	-
Ventilation at index hospital	-	-	-	-	11	0.8
HFNO at index hospital	-	-	-	-	61	4.2
**Comorbidities**						
Hypertension	-	-	-	-	856	59.2
Diabetes	-	-	-	-	675	46.7
Known	-	-	-	-	550	38.0
New diagnosis	-	-	-	-	125	8.6
Chronic obstructive pulmonary disease	-	-	-	-	96	6.6
HIV	-	-	-	-	152	10.5
Cerebrovascular disease	-	-	-	-	62	4.3
Ischemic heart disease	-	-	-	-	86	6.0
Congestive heart failure	-	-	-	-	92	6.3
Chronic kidney disease	-	-	-	-	155	10.7
Dementia	-	-	-	-	58	4.0
Malignancy	-	-	-	-	38	2.6

HFNO, high flow nasal oxygen; HIV, human immunodeficiency virus; s.d., standard deviation; IQR, interquartile range.

### Patient management

Prescription charts were missing or incomplete in 47 (3.3%) of patient records. Less than 25% (*n* = 340) of patients were started on empiric antibiotic therapy for an undifferentiated pneumonia whilst awaiting SARS-CoV-2 polymerase chain reaction (PCR) results at the index hospital. Ceftriaxone combined with azithromycin or amoxicillin-clavulanate was the most commonly prescribed antibiotics (Table 3). A total of 1285 (88.9%) patients received low molecular weight heparin (LMWH) in either prophylactic or therapeutic doses. The majority (*n* = 1154, 79.8%) of patients required oxygen during their stay, with a mean duration of 4.7 days (range 1–30 days). As per protocol after the 19th June 2020, steroids were prescribed for all patients requiring oxygen (*n* = 918, 63.5%).

**TABLE 3:** Prescription data from the index admission through the intermediate care bed facilities admission.

Variables	Mean	s.d.	*n*	%
**Antibiotics**				
Ceftriaxone and/or azithromycin	-	-	280	19.4
Duration of ceftriaxone (days)	3.1	1.6	-	-
Duration of azithromycin (days)	2.8	1.1	-	-
Amoxicillin clavulanate	-	-	91	6.3
Duration (days)	4.0	2.1	-	-
Second line antibiotics	-	-	31	2.1
**LMWH**				
Duration per patient (days)	-	-	-	-
Total LMWH received	8.2	6.4	-	-
Therapeutic dose	6.6	5.1	-	-
Prophylactic dose	7.7	5.4	-	-
Type per patient	-	-	-	-
Prophylactic dosing only	-	-	836	57.8
Therapeutic dosing only	-	-	140	9.7
Prophylactic + therapeutic dosing	-	-	309	21.4
Any LMWH received	-	-	1285	88.9
**Steroids**				
Duration per patient (days)	8.3	5.2	-	-
Prednisone only	-	-	864	59.8
Dexamethasone or hydrocortisone	-	-	271	18.7
Any steroids received	-	-	918	63.5
**Oxygen at ICBF**				
Patients requiring oxygen	-	-	1154	79.8
Duration (days)	4.7	4.2	-	-
Oxygen delivery device during admission,	-	-	-	-
NC/FM/NRB/Combination	-	-	1130	78.1
HFNC	-	-	23	1.6

NC, nasal cannula; FM, face mask; LMWH, low molecular weight heparin; NRB, non-rebreather mask; HFNC, high flow nasal cannula; HFNO, high flow nasal oxygen; s.d, standard deviation.

### Complications

Acute kidney injury (*n* = 283, 19.6%), delirium (*n* = 104, 7.2%) and hypoglycaemia (*n* = 101, 6.9%) were the most common complications, with a lower frequency of diabetic ketoacidosis or hyperosmolar hyperglycaemic state (*n* = 40, 2.8%) and cerebrovascular accident (*n* = 9, 0.6%). Worsening respiratory distress, defined as an increased oxygen demand requiring escalation of oxygen therapy, was observed in 73 patients (5.0%). Facility health care worker COVID-19 infection rate was (16/487, 3.3%).

### Disposition

The mean LOS at the ICBF was 6 days (median 5 days), excluding 20 patients who were transferred out on the day of arrival (Table 4). Overall mortality was 5.7% (*n* = 83) with a median of one death per day (IQR: 0–2) and a maximum of five. The median number of daily patient discharges was 18 (IQR: 11-29). The majority of patients (*n* = 1076, 74.4%) were sent home upon discharge, whilst 83 (5.7%) were transferred out for escalation of care (75 referred to acute care hospitals and eight to intensive care units (ICUs).

**TABLE 4:** Length of stay and disposition.

Variables	Mean ± s.d.	Range	*n*	%
**Length of stay (days)**				
ICBF admission	6.0 ± 4.8	1–41	-	-
Index admission	5.1 ± 5.1	1–41	-	-
**CTICC disposition**				
Home	-	-	1076	74.4
Isolation facility	-	-	196	13.6
Transfers out	-	-	92	6.4
Acute care	-	-	75	5.2
Critical care	-	-	8	0.6
Long term/frail care	-	-	9	0.6
Death	-	-	83	5.7

CTICC, Cape Town International Convention Centre; ICBF, intermediate care bed facilities; s.d., standard deviation.

## Discussion

As of July 2021, South Africa had the highest documented number of COVID-19 cases in Africa and ranked 19th worldwide.^1^ In the course of mitigating the devastating effects of the pandemic, China and many other countries converted large-scale public venues, such as stadiums and exhibition centres, into field hospitals, which were effective in controlling virus transmission and decreasing mortality.^5^ Around the world, COVID-19 field hospitals served different purposes ranging from shelters for isolation of mild COVID-19 patients to specialised COVID hospitals including facilities for ventilation and post-acute care centres.^5,6^

### A unique coronavirus disease in-patient facility – The Cape Town International Convention Centre intermediate care bed facilities in context

The CTICC ICBF provided a unique inpatient setting that differed from other field hospitals. Firstly, we provided hospital-level care including oxygen and did not require patients to provide self-care. Separate isolation facilities (such as existing hotels) housed patients with mild COVID-19 disease (these patients needed to be fully ambulatory and able to perform all aspects of self-care). Secondly, the clinical care plan was adapted to changing health care service needs by adjusting the admission criteria as the patterns of illness shifted. This included the admission of patients from a retirement/frail care facility where an outbreak of COVID-19 had overwhelmed the care capacity; admission of ‘community diabetics’ (diabetic patients with COVID-19 identified by a telemedicine service) who are known to be at higher risk of complications or death from COVID-19,^14,15^ as well as accepting a presumptive clinical diagnosis of COVID-19 when laboratory delays impeded the flow of patients in the health care system. Lastly, palliative care of COVID-19 patients could often not be provided at usual sites of care (e.g. hospice/frail care facilities) because of risks of cross-infection and high bed pressure at acute hospitals. Provision of palliative care was an integral component of the clinical care plan at the CTICC ICBF.

Intermediate care facilities or field hospitals providing surge capacity for infectious disease outbreaks like COVID-19 need to have well considered admission criteria and a clinical care plan that is relevant and matches local resources. This must include clear referral pathways for escalation of care and provision for palliative care. Adaptability and rapid response to the needs of the whole health care service platform through daily communication is a critical success factor. Whilst physical structures may not be able to change rapidly, the systems must allow flexibility to respond to an evolving pandemic. As the operational needs of the health care platform changed in Cape Town, admission criteria were adapted to accommodate the needs through daily communication huddles across the health care platform and within the facility. Through this flexibility, specific vulnerable populations and needs were accommodated. Communication, responsiveness and teamwork were key attributes of the success behind the adaptable referral criteria system: enough flexibility in the types of patients accepted, as well as an organisational culture which favoured team-based learning and service improvement.

### Characteristics, management and outcomes of patients at the Cape Town International Convention Centre intermediate care bed facilities

Our patients have similar demographics to those elsewhere in the world, reflecting the portion of the population severely affected by COVID, especially those with comorbidities.^16,17,18^ Although hypertension is documented as a comorbidity in many studies, there is no evidence to support that patients with hypertension are more susceptible to COVID-19.^19^ Diabetes, however, has a higher rate of complications and mortality in patients with COVID-19.^14^ One striking feature in our sample is the rate of newly diagnosed, and poorly controlled, diabetics. The exceptionally high proportion of poorly controlled and newly diagnosed diabetics in our sample could explain the increased susceptibility of the population of Cape Town to the morbidity and mortality associated with COVID-19.^20^

Although approximately 25% of patients received empiric antibiotics at index hospitals, almost all antibiotics were stopped on arrival at the CTICC ICBF. Concerns emerged that empiric use of antibiotics in the COVID-19 pandemic would lead to antimicrobial resistance because of indiscriminate use.^21^ Data from a local study have shown a low rate of bacterial co-infection in patients with severe COVID-19 and avoiding empiric antibiotics was considered reasonable.^22^ If patients subsequently developed signs or laboratory evidence of a hospital-acquired infection, they were treated with second-line antibiotics.

Specific treatment consisted of LMWH, steroids and oxygen. The prophylactic use of LMWH is associated with lower mortality in COVID-19,^23^ and recommended by clinical guidelines for hospitalised COVID-19 patients.^24^ Most patients only received prophylactic dose LMWH and therapeutic dosing was based on the presumed higher risk in patients with severe/critical illness or elevated D-dimer.

Steroids were prescribed as per provincial health guidelines based on the RECOVERY TRIAL findings.^25,26^ Patients on oxygen received oral prednisone daily unless critically ill (on HFNO) in which case they received dexamethasone or hydrocortisone intravenously according to availability. Overall, 63.5% of patients received steroids, which is less than the eligible 79.8% that received oxygen. This discrepancy is because steroids were only introduced when provincial guidelines came into practice in the third week after opening (end of June 2020). The high rate of hypoglycaemia could be explained by the high number of newly diagnosed and known diabetics as well as the complication of steroid induced hyperglycaemia^27^, which necessitated adjustment in insulin dosages or new initiation in insulin-naïve patients. Modifications to address these hypoglycaemic events included incorporating simpler and safer diabetic sliding scales, specialist input on brittle diabetics and better supervision and support to nursing teams responsible for performing diabetes monitoring and treatment.

Oxygen delivery devices were used in a stepwise manner to provide an increasing fraction of inspired oxygen. Almost 80% of patients needed oxygen (any device) during their admission. Those not requiring oxygen either required specific nursing care, the frail and elderly, or glucose monitoring and management for patients with uncontrolled diabetes. When patients were identified with worsening respiratory distress, and particularly those needing consideration of escalation to HFNO or ventilation, the Western Cape critical care triage tool was used as per guidelines.^12^ Patients eligible for HFNO or ICU admission were identified early, so that they could be transferred to acute care hospitals, as it was not possible to transfer the patient once HFNO was started because of insufficient oxygen capacity on ambulances. A small group of patients^24^ did have HFNO started at CTICC ICBF because they deteriorated and were either awaiting acceptance or transfer to acute care hospitals, or were not eligible for acute hospital care but were judged to be fair candidates for a trial of HFNO at the ICBF. Both local and international studies support the use of HFNO in severe COVID-19 patients outside of ICUs.^28,29^ Although more evidence is needed, HFNO appears to decrease the risk of intubation.^30^ However, the failure rate of HFNO in a South African study was 53%,^28^ and consideration must be given for the limitations of intermediate care settings and ambulance transfer challenges to ICUs.

The mean LOS at the CTICC ICBF was 6 days with a range of 1–41 days. This was slightly higher than that for the index hospital admission (5.1 days). South African modelling data in May 2020 predicted that the duration of hospital stay would be approximately 12 days.^3^ Cape Town International Convention Centre ICBF admission reduced the LOS in acute hospitals by almost 50%. Length of stay is difficult to compare with other countries because of individual hospital admission and discharge criteria. Hospital admission was longer in China than in other countries.^31^ Two field hospitals in convention centres in high income settings reported a median length of stay of 4.6–5.0 days.^32,33^

### Disposition of patients from the intermediate care bed facilities

Some 88% of patients were discharged to home or isolation facilities. This necessitated the inception of designated patient transport service as under lockdown regulations, public transport was restricted. The mortality rate in the ICBF sample (5.7%) was much lower than other published in-hospital mortality rates around the world.^5^ It compares well the overall pooled mortality from 82 countries during the first wave (5.6%).^16^ Higher mortality rates are expected in ICUs and acute hospitals that have more severely ill patients, and the patients accepted by the facility were ideally either past the acute stage of the disease, or deemed to have mild or moderate disease only, other than those accepted specifically for palliative care.

### Utility of field hospitals in the coronavirus disease 2019 and future planning around field hospital capacity

The CTICC ICBF was established with considerable resources from provincial Department of Health funds, in response to modelling, and partly to meet expectations for increasing health care capacity for the COVID-19 pandemic (this being a primary goal of the first ‘lockdown’ phase in South Africa). Despite the best modelling and planning, and setting up an ICBF in a short period of time, the hospital was never utilised beyond 32% of its in-patient capacity. It was also overstaffed for a good deal of its duration, especially as the first wave subsided, and as a second and third ICBF came online in Cape Town by August 2020. Despite the resources spent on the CTICC ICBF, a decision was made to close and dismantle the facility in August 2020. In hindsight, this decision may have been questionable given the subsequent and more severe second wave (Dec 2020 – Feb 2021) and likely subsequent waves, but at this time, it did not seem sustainable to keep the CTICC ICBF open at a high cost, after what appeared to be the conclusion of the pandemic. At the time of the closure, other smaller ICBFs were commissioned (including the 338 bed Brackengate ICBF, situated in a more long-term and economically viable empty industrial warehouse in Cape Town), using many of the operational models and protocols developed at the CTICC ICBF. With the onslaught of the second wave, Cape Town health facilities were once again rapidly overwhelmed, and there was a need to upgrade an existing step-down facility to provide additional beds, but this expenditure was put towards more sustainable facilities rather than short-term temporary structures such as convention centres. This situation was not unique to Cape Town – in the United Kingdom, the Nightingale Hospitals were established but never filled to capacity.^34^ Field hospitals in the United States of America also showed variable rates of utilisation depending on the type of patients accepted and how the field hospital was incorporated into the local health care system.^33^ This is in contrast to the FangCang field hospitals in China which had a high occupancy, likely explained by their use as entry points for triage and quarantine of COVID-19 patients rather than step-down or intermediate care facilities.^5^

### Strengths and limitations

We present what we believe to be the most accurate description of the processes and patient data available, given the constraints of a rapidly established facility during a pandemic. Data collection was limited to that documented in medical records, and the process of organising and scanning records into an electronic system resulted in some missing records. The study population represents a sample selected by admission criteria which may introduce bias in disease severity and outcomes.

## Conclusion

Admission of patients to the CTICC ICBF during the first wave of COVID-19 in the Western Cape province halved the predicted duration of hospital stay which reduced the pressure at acute care hospitals. Utilisation of pre-specified admission criteria and a screening process ensured that the patients admitted were appropriate for intermediate care. Changing pressures necessitated the adjustment of the admission criteria and were safely adopted by communication, responsiveness and teamwork.

This study highlights the role of ICBFs or field hospitals to provide surge capacity during this pandemic and also demonstrated a high level of dedication to providing a standard of care that was comparable to other facilities with regard to patient management. Adaptability and responsivity as well as adequate referral platforms proved to be crucial to allow this. Rapid responsivity to the needs of the whole health care service platform through daily communication was also highlighted as a critical success factor and should be a built-in feature. Despite the low bed utilisation of the ICBF, its strengths in design, implementation and flexibility will guide more cost-effective use of ICBFs during this pandemic. This study will assist future health planning and strategy development with regard to ICBFs and their role in this current pandemic and future disease outbreaks.
